# Automated radiotherapy planning for volumetric modulated arc therapy in lung cancer

**DOI:** 10.1002/acm2.70297

**Published:** 2025-10-10

**Authors:** Johann Brand, Juliane Szkitsak, Bernd‐Niklas Axer, Lucas Pieper, Oliver J. Ott, Marlen Haderlein, Florian Putz, Rainer Fietkau, Christoph Bert, Stefan Speer

**Affiliations:** ^1^ Department of Radiation Oncology Universitätsklinikum Erlangen Friedrich‐Alexander‐Universität Erlangen‐Nürnberg (FAU) Erlangen Germany; ^2^ Comprehensive Cancer Center Erlangen‐EMN (CCC ER‐EMN) Erlangen Germany; ^3^ Bavarian Cancer Research Center (BZKF) Erlangen Germany; ^4^ Siemens Healthineers AG Forchheim Germany

**Keywords:** automated treatment planning, lung cancer, VMAT volumetric modulated arc therapy

## Abstract

**Background:**

Volumetric‐modulated arc therapy (VMAT) treatment planning balances the need for adequate coverage of the planning target volume (PTV) and the sparing of organs‐at‐risk (OARs). However, this time‐consuming iterative process is influenced by the planner's experience, personal preferences, and the time devoted to create the plan. This often leads to a considerable variability in plan quality.

**Purpose:**

For lung tumors, where PTV size and the relative location between OARs and PTV vary widely, these challenges are particularly pronounced. This work aims to develop an automated treatment planning solution for lung tumors, standardizing the process and ensuring consistent, high‐quality plans while significantly reducing the planner's active workload and time investment

**Methods:**

An automated treatment planning software, named Uniklinikum Erlangen‐Automated Treatment Planning (UKER‐ATP), developed within the RayStation (RaySearch, Stockholm, Sweden, Version 12A) treatment planning system using its Python interface, was employed to automate the entire planning process. This software combines both scripted and knowledge‐based methods; for the latter, overlap‐z‐histogram (OZH) and overlap volume histogram (OVH) were used to predict dose volume histograms (DVHs). This study included 15 clinical lung cancer patients with manually created VMAT treatment plans as part of their therapy. For each patient, an automated plan (AP) was generated and compared with the manual plan (MP) created by physicists in our institute. Dosimetric parameters and plan quality indices were evaluated. Furthermore, four board‐certified physicians conducted a direct comparison of the plans to assess quality.

**Results:**

The APs achieved comparable coverage of the PTV while demonstrating improved dose conformity and uniformity compared with the MPs. Mean dose and $V_{20\;\mathrm{Gy}}$ of the total lung were significantly lower in the APs compared with those in the MPs ($p_{\mathrm{mean}\;\mathrm{dose}}$ < 0.01, $p_{V_{20\;\mathrm{Gy}}}$ = 0.01 respectively); the mean dose of the heart was also significantly reduced in the APs (p < 0.05). Furthermore, APs presented less variability in DVH metrics. 57% of the AP plans were rated by physicians as superior to their manually created counterparts, and 78% were rated as either superior or equivalent. In most cases, MPs selected as superior were created by highly experienced planners, whereas APs were consistently preferred when MPs had been created by less experienced planners. This trend is supported by a significant negative between planner experience and AP superiority (Spearman's ρ = –0.541, p = 0.037), suggesting that APs tend to outperform MPs when planner experience is limited.

**Conclusion:**

Our automated VMAT plan creation software, especially designed for lung tumors, effectively achieves target coverage while minimizing doses to OARs. Despite the complexities associated with lung tumors, such as variable PTV sizes and OAR locations, the software demonstrated robust performance.

## 1 INTRODUCTION

In the past few decades, treatment planning in radiotherapy has made considerable progress due to innovative hardware and software solutions, leading to modalities such as intensity‐modulated radiation therapy^1^, ^2^, ^3^ and volumetric‐modulated arc therapy (VMAT),^4^, ^5^, ^6^ which significantly improve treatment outcomes.^7^ However, treatment planning became increasingly complex and therefore more time consuming.^8^

Treatment planning is a compromise between a sufficient coverage of the planning target volume (PTV) and a simultaneous dose sparing of organs‐at‐risk (OARs). However, this is particularly challenging for complex OAR‐PTV geometries. It is difficult to decide whether it is possible or worth it spending more time to further spare surrounding organs. As such, the quality is dependent on the planner's level of experience, preferences, and the amount of time they invest in the planning process,^9^, ^10^, ^11^, ^12^ leading to a high variability between planners.

These variabilities drive a shift towards automated planning techniques, ensuring consistent and high‐quality plans. The most common methods of automatic planning are currently knowledge‐based planning,^13^, ^14^ deep learning approaches,^8^, ^15^, ^16^, ^17^, ^18^ protocol‐based iterative optimization,^9^, ^12^, ^19^, ^20^, ^21^ as well as multi‐criteria optimization.^22^, ^23^ However, studies on knowledge‐based and protocol‐based planning with Raystation (RaySearch, Stockholm, Sweden)^24^, ^25^ treatment planning system (TPS) are still rare, particularly for lung tumors.^26^, ^27^

Therefore, a novel approach to automate lung cancer VMAT planning combining a knowledge‐based structure dependent dose volume histogram (DVH) metric prediction module and protocol‐based planning, named Uniklinikum Erlangen‐Automated Treatment Planning (UKER‐ATP), is presented in this work.

Its performance was evaluated using dosimetric parameters and plan quality indices. Furthermore, four experienced radiation oncologists conducted a direct comparison between the automated plans (APs) and manual plans (MPs). Beyond overall plan quality, the study also examined how planners’ experience influenced physicians’ preferences during plan selection.

## 2 METHODS

**FIGURE 1:**
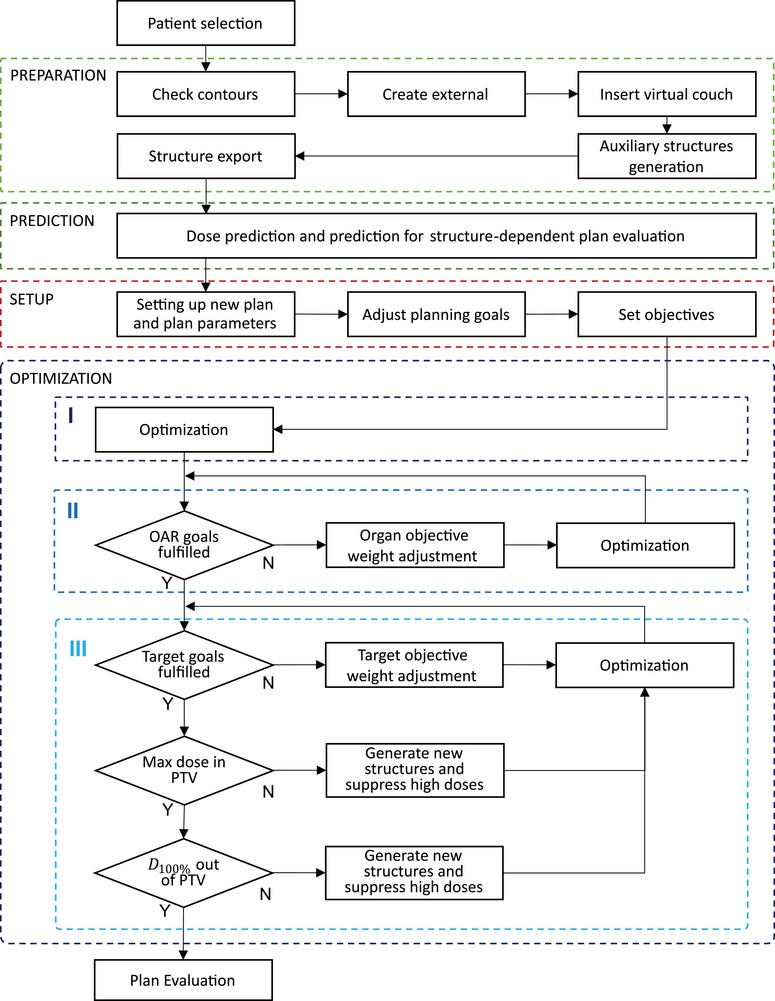
The workflow of UKER‐ATP comprises four modules: preparation, prediction, setup, and optimization. The optimization module is further divided into three subgroups: “preliminary optimization” (I), “organ‐at‐risk optimization” (II), and “target optimization” (III).

UKER‐ATP was developed based on the treatment planning system RayStation (RaySearch, Stockholm, Sweden, version 12A) and its scripting capabilities using the Python (version 3.8) environment. UKER‐ATP is specifically designed for lung tumors and allows the planner to enter their preferences via a graphical user interface (GUI). It consists of four modules corresponding to the four phases of planning: preparation, prediction, setup, and finally optimization. The structure of UKER‐ATP and the detailed workflow are shown in Figure 1, details of each module are described in this section.

### 2.1 Patient and image dataset

To develop the predictive capabilities of the UKER‐ATPs prediction module, a basis of 103 lung tumor patients was used. All patients received standard treatment in our department, which included PET‐CT‐based definition of the PTV, encompassing the primary tumor region and lymph node metastases, each surrounded by a safety margin of 5–10 mm. In the initial phase, the defined PTV was irradiated with a total dose of 50.4 Gy delivered in 28 fractions. If tumor shrinkage permitted, the target volume was subsequently adapted, and a dose escalation (boost) was applied up to a total of 66.6 Gy.

For the prediction module, treatment plans covering the first 28 radiotherapy fractions were used. All patients were treated with VMAT as part of the standard clinical workflow between 2019 and 2024. This resulted in a variety of PTV locations within the lung, planned by a total of 15 different medical physicists. Additional constraints included a prescribed dose of 50.4 Gy delivered in 28 fractions using a 6 MV photon beam. All treatment plans were consistently generated with two full arcs. The average size of the PTV was 633 cm^3^ (range: 85 cm^3^–1672 cm^3^). A mean PTV coverage of 94.6% was achieved for all patients within the cohort. At the time of treatment, the patients were between 41 and 84 years old (mean age: 66.0 years). The cohort is subdivided into 43 female and 60 male patients.

For each patient plan, the approved RT structure set, RT‐dose and RT‐plan were exported from RayStation. The data was collected in a database independent from the treatment planning system.

### 2.2 Preparation module

The preparation module consists of five individual subgroups (see Figure 1 preparation). The first step is performed automatically in the background, ensuring that the regions of interest (ROIs) are consistently named and ROIs required for planning available. To ensure name consistency and correct identification of the ROIs, a dictionary was created based on the naming conventions used at our center. The module automatically identifies each ROI by referencing this name dictionary.

**FIGURE A1:**
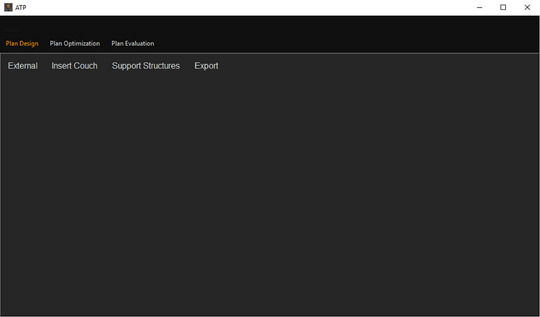
The GUI of UKER‐ATP at the PREPARATION phase. The planner is guided from left to right through the buttons “External,” “Insert Couch,” “Support Structures,” and “Export,” which handle the individual tasks of the preparation phase.

Guided through a GUI, the planner first creates an external structure, if not already available, followed by inserting a virtual couch. Additional auxiliary ROIs like the skin, an extended esophagus structure and a structure for minimizing excessive anterior irradiation are generated using algebraic functions. Subsequently, the planner is prompted via the GUI to export the structure set, which is then used in the background outside of RayStation to perform the dose prediction. In Figure A1 of the appendix, the interface of the GUI at this stage of the preparation phase is shown.

### 2.3 Prediction module

In general, knowledge‐based planning systems predict DVHs by learning from a large number of clinically accepted plans.^13^ UKER‐ATP incorporates this concept using a geometry‐driven approach to estimate patient‐specific, achievable DVH metrics. Central to this approach is the quantification of the spatial relationship between the PTV and OARs, using two complementary descriptors: the overlap volume histogram (OVH) and the overlap‐z‐histogram (OZH).

The OVH, originally introduced by Wu et al.,^29^ describes the geometric relationship between a PTV and an OAR based on their volumetric overlap. While this method has proven useful, it has certain limitations: due to its rotational symmetry, it cannot adequately capture spatial relationships along the cranio‐caudal axis.^28^

To address this limitation, the OZH was introduced,^28^ capturing the overlap between PTV and OAR along the cranio‐caudal direction. By combining both OVH and OZH, a geometric profile of the anatomical configuration is obtained, forming the basis of the UKER‐ATP prediction module.

This module comprises two components: a point prediction of DVH metrics values, providing a single‐value, and an interval prediction that accounts for inter‐patient planning variability. Both components are executed externally from the RayStation treatment planning system.

#### 2.3.1 Point prediction

The prediction is based on the algorithm described by Brand et al.,^28^ which identifies previously treated patients with similar OVH and OZH profiles. After exporting the structure set of a new patient, the OVH and OZH are computed and compared to those in the previously described patient and image dataset. The algorithm searches for the five best‐matching cases based on combined OVH–OZH similarity. The DVH curves of the matched patients are used to extract the relevant DVH metric values, which are then averaged to generate the predicted single‐value for the new patient. The system consistently applies this prediction approach to a fixed set of OARs: heart, spinal cord, esophagus, total lung, and left and right breast. The specific DVH metric types predicted for these structures are listed in Table 1.

**TABLE 1:** Predicted planning objectives for critical structures. $V_{x}$ represents the relative volume of an organ receiving a dose of x Gy, $D_{x}$ denotes the dose delivered to the hottest x cubic centimeters (cc) or percent of a specified volume.

OARs	Metric type
Total lung	Mean (Gy)
	$V_{20\;\mathrm{Gy}}$ (%)
Heart	Mean (Gy)
Spinal cord	$D_{0.01\;\mathrm{cc}}$ (Gy)
Esophagus	$D_{0.5\;\mathrm{cc}}$ (Gy)
Skin	$D_{0.5\;\mathrm{cc}}$ (Gy)
Left breast	Mean (Gy)
Right breast	Mean (Gy)

#### 2.3.2 Interval prediction

In addition to point predictions, the system offers interval predictions that reflect the variability inherent in clinical planning. This robust estimation method uses the area under the curve (AUC) of both OVH and OZH as predictive features. The interval model is determined upon the patients within the previously described patient and image dataset.

**FIGURE 2:**
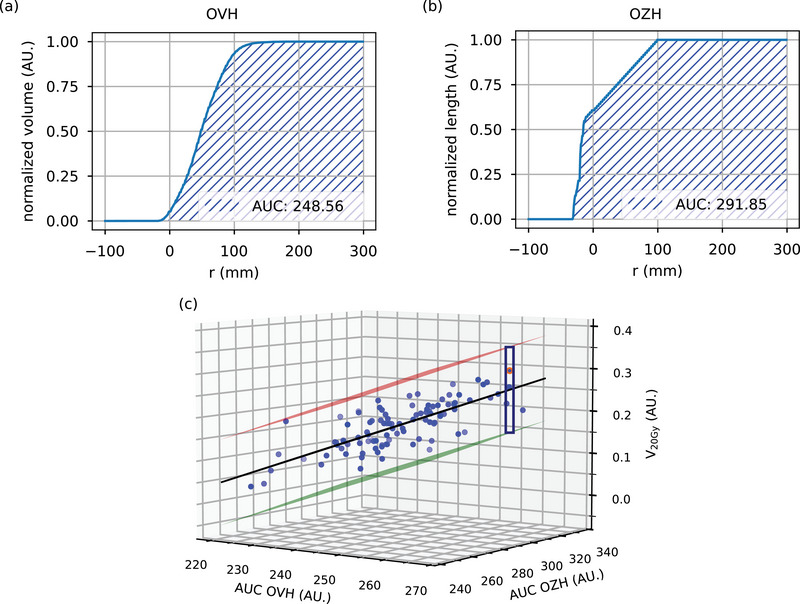
(a) and (b) show the calculation of the AUC from the OVH and OZH of the total lung for an exemplary patient. (c) illustrates the relationship between AUC(OVH), AUC(OZH), and the corresponding $V_{20\;\mathrm{Gy}}$ value, modeled by a regression plane fitted to all 103 patients in the dataset. The orange marker indicates the example patient shown in (a) and (b). The prediction interval for $V_{20\;\mathrm{Gy}}$ is obtained by shifting the regression plane by $3\sigma_{res}$. For the highlighted patient, the interval ranges from 0.19 to 0.39, shown by the blue box.

Figure 2a,b illustrate how the area under the curve (AUC) is computed from the OVH and OZH of the total lung for an exemplary patient. This patient, highlighted in orange in Figure 2c, serves to demonstrate the application of the model. Figure 2c depicts the relationship between AUC(OVH), AUC(OZH), and the corresponding $V_{20\;\mathrm{Gy}}$ value, which is used here as a representative DVH metric. A regression plane was fitted to the data from all patients from the dataset. As shown in Figure 2c, by shifting the fitted plane upward and downward by three times the residual standard deviation ($3\sigma_{\mathrm{res}}$), an interval can be established for $V_{20\;\mathrm{Gy}}$ with a 99.73% confidence. For the patient plan marked in orange, the interval prediction yields a $V_{20\;\mathrm{Gy}}$ range from 0.19 to 0.39, as indicated by the blue box in Figure 2c.

This modeling approach is not limited to $V_{20\;\mathrm{Gy}}$ of the total lung but extends to other DVH metrics and OARs as well. For $\bar{D}$_Lung_ of the total lung, $\bar{D}$_Heart_, and $\bar{D}$_Breast_, three dimensional linear relationships were identified between the AUC of OVH, AUC of OZH, and the corresponding DVH metric value. The $D_{0.5\;\mathrm{cc}}$ of the esophagus and the $D_{0.5\;\mathrm{cc}}$ of the skin exhibit a correlation with the AUC of the OVH, without considering the OZH. In these cases, the interval prediction is constructed analogously to the three‐dimensional models by shifting the fitted regression line upward and downward by $3\sigma_{\mathrm{res}}$. The $D_{0.01\;\mathrm{cc}}$ of the spinal cord is independent of both OVH and OZH, here, the prediction interval is defined by 3 times the standard deviation of the observed distribution.

The predicted interval bounds are utilized in both the setup module and optimization module modules. In the setup module, the lower and upper bounds define an expected achievable range. In the optimization module, these bounds are used for feature normalization: each DVH metric value is linearly scaled to the interval [0,1], where a value of 1 corresponds to the lower bound (representing optimal sparing) and 0 to the upper bound (representing the least favorable outcome within the predicted range). This normalized scale facilitates consistent weighting and comparison of OAR objectives within the multi‐objective optimization, based on patient‐specific anatomical constraints.

### 2.4 Plan setup module

**FIGURE A2:**
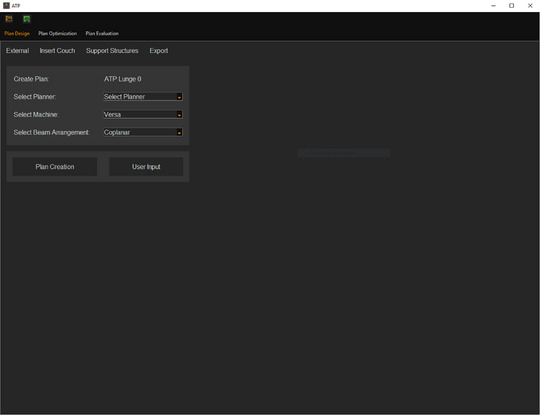
The graphical user interface of UKER‐ATP during the SETUP phase. This interface appears after the PREDICTION module and enables the planner to generate a treatment plan based on predicted objective values. The planner can select a linear accelerator and configure the beam configuration to optimize treatment delivery.

Once the prediction module is completed, the UKER‐ATP graphical user interface (GUI) can be re‐opened. The predictions are automatically imported into the system, enabling the planner to proceed to the next step: the setup module (see Figure 1). An example of the GUI interface at this stage is shown in Appendix Figure A2.

In the SETUP module, the planner configures the treatment plan. This involves configuring the plan parameters, including the planner's name, selecting the linear accelerator, and setting the maximum monitor units (MUs) per fraction per arc to 350 MU. This value is an empirically derived parameter used at our institute to manage plan complexity, particularly for single doses of 1.8–2 Gy delivered with two full arcs. The planner also specifies whether the treatment will use planar or co‐planar beam arrangements. In both scenarios, a pre‐defined beam arrangement is automatically generated. For this study, only co‐planar beam arrangements were utilized, consisting of two full arcs, as the database primarily contains co‐planar plans from previous cases. Subsequently, the results of the prediction module are applied.

**FIGURE 3:**
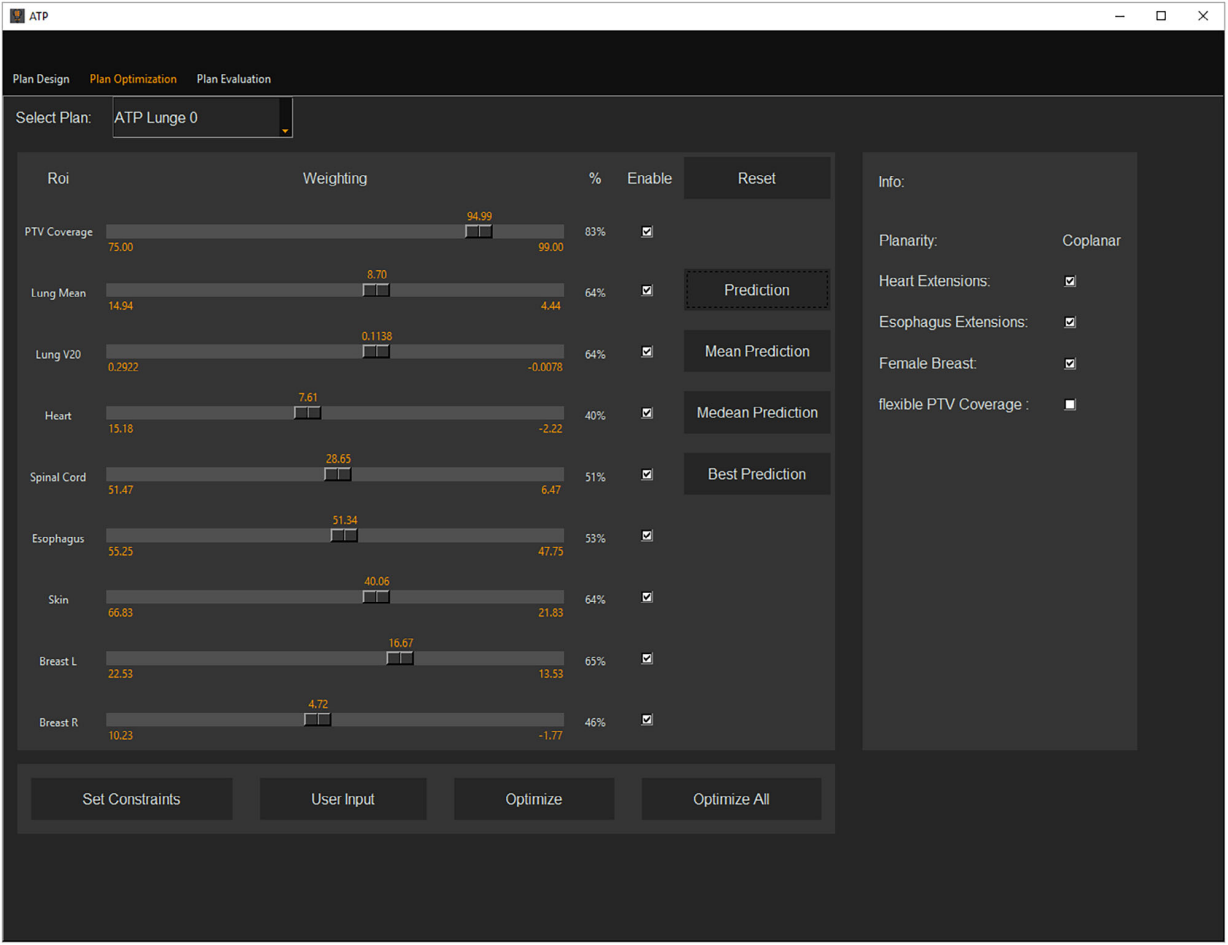
The GUI of the UKER‐ATP system within the setup module. The interface allows planners to configure plan parameters, adjust objective values for optimization, and select whether auxiliary structures, such as heart and esophagus extensions, are taken into account. Predictions from the prediction module provide initial objective values, which can be fine‐tuned using sliders within predefined boundaries.

Based on the point and interval predictions, UKER‐ATP determines planning goals for optimization, which can be manually adjusted using sliders within the upper and lower boundaries of the interval prediction, allowing flexibility based on the planner's preference (see image of the GUI, Figure 3). According to these planning goals, UKER‐ATP generates objectives for the OAR metrics listed in Table 1. For the PTV, objectives are created for minimum dose, maximum dose, and uniform dose. By default, the weights of the objectives are set to 10 for OARs and 100 for the PTV. To reach the planning goals, the actual objective values are set 10% lower than the planning goals, allowing the optimizer sufficient margin to meet the targets during optimization.

### 2.5 Optimization module

Similar to script‐based automated planning algorithms, the optimization module of UKER‐ATP follows the natural workflow of an experienced planner. As shown in Figure 1d–f, the optimization module consists of three interconnected submodules: “preliminary optimization”, “organ‐at‐risk optimization”, and “target optimization”.

The “preliminary optimization” (see Figure 1, optimization I) conducts an initial evaluation to quantify the deviation between the planned target metrics and the actual results obtained after the first optimization step. This deviation, referred to as the value gap $\Delta_{i}=T-A_{i}$, is defined as the difference between the set planning goal T and the current DVH metric value $A_{i}$ after optimization i. Both T and $A_{i}$ are normalized using the interval bounds predicted by the interval prediction of the prediction module. Additionally, the process evaluates the objective function value $F_{i}^{\mathrm{obj}}$ of each individual planning objective, and the total objective function value across all objectives $F_{i}^{\mathrm{tot}}$ used by the TPS during optimization. These values form the basis for iterative weight adjustment.

In “organ‐at‐risk optimization” (see Figure 1, optimization II), the algorithm updates the weight $w_{i}$ of the current iteration i of each OAR objective based on the value gap $\Delta_{i-1}$ and the relative contribution $F_{i}^{\mathrm{tot}}/F_{i}^{\mathrm{obj}}$ of the prior iteration i‐1. The updated weight $w_{i}$ is calculated as:

(1) $$\begin{array}{cc} & w_{i}^{\mathrm{OAR}}\left(F_{i-1}^{\mathrm{tot}},F_{i-1}^{\mathrm{obj}},\Delta_{i-1}\right)\\ & =\left\{\begin{array}{c} w_{i-1}\cdot 0.001,if\;\Delta_{i-1}\leq -0.999\\ w_{i-1}\cdot \left(1+\Delta_{i}\right),if-0.999<\Delta_{i-1}<-0.05\\ w_{i-1}\cdot \left(1+0.1\cdot \frac{F_{i-1}^{\mathrm{tot}}}{F_{i-1}^{\mathrm{obj}}}\right),if\;\Delta_{i-1}>0\;and\;\frac{F_{i-1}^{\mathrm{tot}}}{F_{i-1}^{\mathrm{obj}}}<100\\ w_{i-1}\cdot 11,if\;\Delta_{i-1}>0\;and\;\frac{F_{i-1}^{\mathrm{tot}}}{F_{i-1}^{\mathrm{obj}}}\geq 100 \end{array}\right. \end{array}$$

The process is repeated iteratively until all OAR objectives are fulfilled. In “target optimization” (see Figure 1, optimization III), the algorithm focuses on achieving sufficient PTV coverage while suppressing high‐dose regions outside the target. This is done by adjusting the weights of both the PTV minimum dose and PTV maximum dose objectives, depending on the value gap $\Delta_{i-1}$. The update rule is defined as:

(2) $$\begin{array}{cc} & w_{i}^{\mathrm{PTV}}\left(F_{i-1}^{\mathrm{tot}},F_{i-1}^{\mathrm{obj}},\Delta_{i-1}\right)\\ & \;=\left\{\begin{array}{c} w_{i-1}\cdot \left(1+0.1\cdot \frac{F_{i-1}^{\mathrm{tot}}}{F_{i-1}^{obj}}\right),if\;0<\Delta_{i-1}<0.1\\ w_{i-1}\cdot \left(1+0.2\cdot \frac{F_{i-1}^{\mathrm{tot}}}{F_{i-1}^{\mathrm{obj}}}\right),if\;\Delta_{i-1}\geq 0.1 \end{array}\right. \end{array}$$

In parallel to this target weight adaptation, a dedicated suppression mechanism is applied to penalize high‐dose regions outside the PTV and ensure the max dose to be within in the PTV. In each iteration i, an auxiliary ROI is automatically generated to capture the volume receiving at least 95% of the prescription dose but not overlapping with the PTV. A maximum dose objective is created with the weight:

(3) $$w^{\mathrm{high}-\mathrm{dose}}\left(i\right)=100\cdot i$$

where i denotes the current number of high‐dose suppressions within the “target optimization”. After each iteration, the auxiliary ROI is deleted and re‐generated based on the updated dose distribution.

The complete optimization process, encompassing “preliminary optimization”, “organ‐at‐risk optimization” and “target optimization” are performed iteratively until all constraints are satisfied or a maximum of 40 iterations in total is reached. If a feasible solution is not found within this limit, the planner is prompted to revise the planning goals manually.

### 2.6 Evaluation and analysis

Fifteen clinically used patient plans, which were not included in the database for the prediction module, were chosen to independently evaluate the quality of plans generated by UKER‐ATP (AP) against the manually optimized counterparts (MP). The structure sets used for the MPs were also employed for the APs to ensure comparability. Of these fifteen patients, six patients are female (40%) and nine patients are male (60%) with a median age of 64 years and an age range of 42 to 80 years. The according treatment plans were created by eight different physicists with varying levels of experience. Planner experience was defined as the cumulative number of finalized treatment plans per physicist, extracted from MOSAIQ Oncology Information System (Version 2.81, Elekta AB, Stockholm, Sweden), and reflects all plans generated up to the time of plan creation, irrespective of tumor site (see Table 2). Detailed information about the patients and associated tumors are provided in Table 2.

**TABLE 2:** Patient collective (N = 15) details, including gender distribution as female (F) and male (M).

Patient Plan	Age	Sex	Cancer type	Tumor stage	Tumor site	Volume PTV [cm^3^]	Total lung prox. [mm]	Heart prox. [mm]	Spinal cord prox. [mm]	Esophagus prox. [mm]	Experience
1	61	M	NSCLC	IVB	Medi	443.20	−23	−17	14	−24	818
2	55	F	NSCLC	IIIC	Medi	604.17	−15	−21	14	−15	384
3	66	F	NSCLC	IIIA	RUL + Medi	352,54	−27	−16	17	−4	104
4	80	M	NSCLC	IIIA	RL	655,21	−27	−19	14	−15	1371
5	65	F	NSCLC	IIIA	Medi	540.24	−15	−15	11	−17	65
6	60	M	SCLC	ED	LUL	1149.66	−30	−23	6	−29	681
7	67	F	NSCLC	IVA	LUL	273.59	−24	−6	29	−5	117
8	52	M	NSCLC	IIIC	RUL + Medi	1417.07	−21	−23	2	−24	806
9	64	M	NSCLC	IIIC	LUL+ Medi	547.88	−24	−12	1	−21	25
10	53	M	NSCLC	IIIB	LL	1400.00	−24	−24	1	−16	1063
11	42	M	SCLC	LD	Medi	826.17	−22	−27	14	−20	1056
12	72	F	SCLC	LD	Medi	303.05	−16	−12	14	−20	167
13	65	M	NSCLC	IIb	Medi	463.67	−18	−21	11	−16	153
14	66	F	SCLC	LD	Medi	108.99	−11	2	33	−3	298
15	55	M	SCLC	LD	Medi	410.18	−16	−21	9	−8	155

*Note*: Lung tumor sites were categorized into the mediastinum (Medi), right lung (RL), and left lung (LL), with further subdivisions into the right upper lobe (RUL), right middle lobe (RML), right lower lobe (RLL), left upper lobe (LUL), and left lower lobe (LLL). Tumor stage is reported according to the Union for International Cancer Control (UICC) classification 8th edition for non‐small cell lung cancers (NSCLCs) and as limited disease (LD) or extensive disease (ED) for small cell lung cancers (SCLCs). Prox. denotes the proximity between the tumor volume and the respective organ: Positive values represent the minimal distance, while negative values signify penetration of the tumor volume into the organ. The column Experience denotes the number of treatment plans the planner has generated since 2020 up to the time of plan creation, irrespective of the tumor site.

In addition to the OAR metrics used for prediction and plan optimization (see Table 1), the following metrics were evaluated to compare the quality of the APs and MPs for the PTV: $D_{2\%}$, $D_{50\%}$, $D_{98\%}$, Coverage (C), Homogeneity Index (HI),^30^ and Conformity Index (CI).^31^

The coverage was defined as:

(4) $$C=\frac{TV_{\mathrm{RI}}}{\mathrm{TV}}$$

$TV_{\mathrm{RI}}$ is the target volume covered by the reference isodose (95% of the prescription dose) and TV is the volume of the target.

The HI was defined as:

(5) $$\mathrm{HI}=\frac{D_{2\%}-D_{98\%}}{D_{p}}$$

where $D_{p}$ represents the prescription dose. A value of HI closer to 0 indicates better dose homogeneity. CI was calculated as:

(6) $$\mathrm{CI}=\frac{TV_{\mathrm{RI}}}{\mathrm{TV}}\cdot \frac{TV_{\mathrm{RI}}}{V_{\mathrm{RI}}}$$

where $TV_{RI}$ is the target volume (PTV) covered by the reference isodose (RI, 95%), TV is the total target volume, and $V_{RI}$ is the total volume covered by the reference isodose. CI ranges from 0 to 1, with the ideal value of 1 representing perfect conformity. The differences in dose metrics were tested for statistical significance using the Wilcoxon matched‐pair signed‐rank test, with a significance level of 5%.

Furthermore, four senior radiation oncologists, with an average of 23 years of professional experience, independently conducted a blinded review of all APs and MPs. In the following, they are referred to as physicians 1–4. Physicians 1 and 4 had extensive expertise in stereotactic lung treatments, physician three specialized in conventional lung radiotherapy, and physician two was a highly experienced general radiation oncologist. Their evaluation was based on dose distributions and DVHs for all relevant structures. The first step was to determine whether each plan met the criteria for clinical acceptability. Subsequently, the oncologists conducted a blinded evaluation to determine which plan was superior or whether both were equivalent. To assess the consistency of their evaluations, Fleiss' kappa was analyzed for all possible combinations of oncologists to evaluate inter‐clinician agreement across multiple raters, with agreement levels categorized as excellent (κ > 0.81), good (0.61–0.80), moderate (0.41–0.60), fair (0.21–0.40), or poor (κ < 0.20).

Subsequently, Spearman's rank correlation coefficient was used to analyze potential correlations between planning experience (see Table 2) and the oncologists' evaluations. The Spearman's rank correlation coefficient (ρ) ranges from −1 to 1, where *r* = 1 indicates a perfect positive correlation, meaning that higher planning experience is consistently associated with better evaluations. Conversely, ρ = −1 represents a perfect negative correlation, indicating that greater planning experience corresponds to lower evaluations. A value of ρ = 0 suggests no correlation between planning experience and oncologists' assessments.

Plan‐specific quality assurance (QA) was conducted for all MPs and APs using the ArcCHECK^32^ device (Sun Nuclear Corporation, Melbourne, USA) to assess the clinical deliverability of the treatment plans. This device features a cylindrical, water‐equivalent phantom embedded with a 3D array of 1386 diode detectors arranged in a spiral pattern with 10 mm spacing between sensors. Measured doses were compared to treatment planning system calculations using gamma analysis with the software SNC patient (Sun Nuclear Corporation, Melbourne, USA, version 6.6). A global γ‐criteria of 3%/3 mm for dose difference and distance‐to‐agreement was applied. QA verification was deemed successful if the γ‐passing rate exceeded 95%. Consistency was ensured by performing all measurements under identical setup conditions on the same day.

To assess treatment delivery efficiency, MUs for all plans were analyzed. The Modulation Complexity Score (MCS) was calculated to assess the clinical feasibility of MPs and APs. MCS ranges from 0.0 to 1.0, where lower values indicate higher modulation complexity, meaning the plan has more intricate variations in fluence and is potentially more challenging to deliver accurately.^33^ Additionally, Leaf Travel (LT) was analyzed to evaluate the mechanical complexity of the treatment plans. LT measures the total distance traveled by the multi‐leaf collimator during treatment delivery, where higher values may indicate increased modulation and potentially prolonged delivery times.^34^ Both, MCS and LT were calculated using UCoMX.^35^, ^36^ Differences between MPs and APs, including the mentioned metrics, gamma analysis outcomes, and MU comparisons, were statistically evaluated using the Wilcoxon signed‐rank test. A significance level of p < 0.05 was used to determine statistical significance.

## 3 RESULTS

### 3.1 DVH metric analysis

The dosimetric comparison between APs and MPs was evaluated using DVH metrics for the PTV and OARs. The results, reported as mean ± standard deviation (SD) in Table 3, showed statistically significant differences in some parameters. For the PTV, significant differences were observed in $D_{2\%}$ and $D_{50\%}$ metrics (p = 0.01 and p < 0.01, respectively). The APs demonstrated a slightly lower $D_{2\%}$ (52.22 ± 0.38 Gy) compared to MPs (52.58 ± 0.39 Gy, p = 0.01) and a similarly lower $D_{50\%}$ (50.29 ± 0.17 Gy for APs vs. 50.51 ± 0.18 Gy for MPs, p < 0.01). However, no significant difference was found for $D_{98\%}$ (46.69 ± 0.77 Gy for APs vs. 46.58 ± 1.06 Gy for MPs, p = 0.34). The HI was slightly better in APs (0.11 ± 0.017) compared to MPs (0.12 ± 0.023, p < 0.01), indicating improved dose uniformity. Conversely, the conformity index and coverage metrics showed no statistically significant differences (p = 0.45 for CI and p = 0.68 for C).

**TABLE 3:** Comparison of dosimetric parameters between automated and manual plans for the PTV and OARs, reported as mean ± standard deviation (SD).

	Metric	APs (mean ± SD)	MPs (mean ± SD)	p‐Values
PTV	$D_{2\%}$ (Gy)	52.22 ± 0.38	52.58 ± 0.39	0.01
	$D_{50\%}$ (Gy)	50.29 ± 0.17	50.51 ± 0.18	<0.01
	$D_{98\%}$ (Gy)	46.69 ± 0.77	46.58 ± 1.06	0.34
	HI	0.11 ± 0.017	0.12 ± 0.023	<0.01
	CI	0.85 ± 0.041	0.80 ± 0.16	0.45
	C	94.67 ± 1.90	94.84 ± 2.46	0.33
Total lung	Mean (Gy)	11.98 ± 3.18	12.34 ± 3.23	<0.01
	$V_{20\;\mathrm{Gy}}$ (%)	18.32 ± 5.95	19.41 ± 6.17	0.01
Heart	Mean (Gy)	12.74 ± 6.85	13.19 ± 7.19	0.05
Spinal cord	$D_{0.5\;\mathrm{cc}}$ (Gy)	27.69 ± 3.40	27.59 ± 5.13	0.64
Esophagus	$D_{0.01\;\mathrm{cc}}$ (Gy)	50.12 ± 2.62	50.85 ± 2.12	0.02

*Note*: The results show statistically significant differences for all lung and heart parameters, as well as for $D_{50\%}$ and HI of the PTV.

**FIGURE 4:**
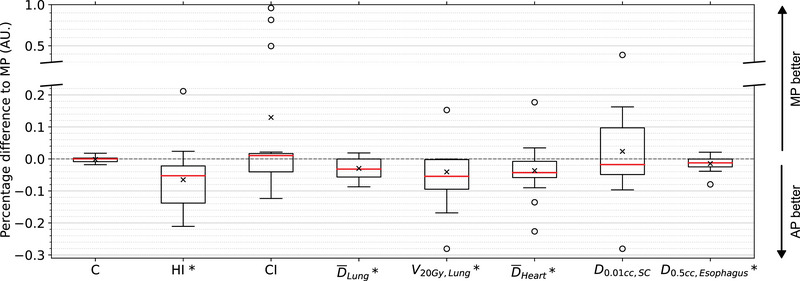
Boxplots illustrating the differences in key dosimetric parameters between APs and MPs. Negative differences indicate that the values for APs are smaller compared to MPs. The edges of the box represent the 25th and 75th percentiles, with red lines indicating the median. The mean values are marked as ‘x’. Black circles denote outliers. The spinal cord is abbreviated as “SC” A star (*) indicates statistically significant differences with a p‐value of 0.05 or lower.

For the OARs, the mean lung dose was significantly lower in APs (11.98 ± 3.18 Gy) compared to MPs (12.34 ± 3.23 Gy, p < 0.01). Similarly, the lung $V_{20\;\mathrm{Gy}}$ was significantly reduced in APs (18.32 ± 5.95%) compared to MPs (19.41 ± 6.17%, p = 0.01). For the heart, the mean dose was slightly lower in APs (12.74 ± 6.85 Gy) compared to MPs (13.19 ± 7.19 Gy), with statistical significance (p = 0.05). Regarding the esophagus, the $D_{0.5cc}$ was significantly lower in APs (50.12 ± 2.62 Gy) than in MPs (50.85 ± 2.12 Gy, p < 0.02). No significant differences were observed in the spinal cord $D_{0.01\;\mathrm{cc}}$ metric 27.69 ± 3.40 Gy for APs vs. 27.59 ± 5.13 Gy for MPs). Figure 4 shows the boxplots of relative percentage differences in dosimetric parameters for the OARs between APs and MPs. It is evident that the dose in all OARs is lower in both the mean and median values for APs compared to MPs, with the exception of the spinal cord.

### 3.2 Clinical analysis

**FIGURE 5:**
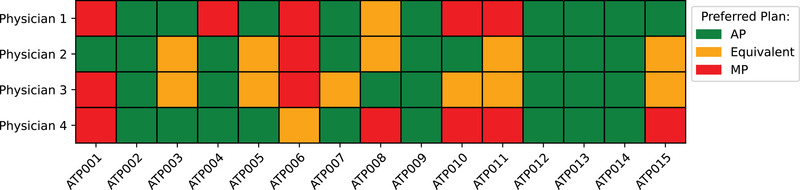
Preference matrix of the four different experts. The matrix highlights differences and agreements in plan assessments. Preference evaluation of four physicians (Physician 1–4) for 15 treatment plans (Plan 1–15). Colors indicate preferences: Red (MP), Yellow (Equivalent), and Green (AP).

Figure 5 presents the preference matrix summarizing the ratings of the four physicians across the 15 plans. The results reveal a diverse set of preferences. Physician 1 and 4 demonstrated a balanced evaluation, with favoring AP. Physician 3 exhibited a relatively even distribution of preferences across AP, MP, and equivalence. Physician 2 leaned toward AP in most cases, with fewer instances of preference for MP or equivalent ratings.

Out of the 60 evaluations, AP was preferred in 34 cases (≈56.7%), while MP was chosen in 13 cases (≈21.3%), and 13 cases (≈21.3%) were rated as equivalent. Examining individual preferences, Physicians 1, 2, and 4 each preferred AP in 60% of cases, whereas Physician 3 showed the lowest preference for AP, selecting it in only 46.7% of cases. MP was particularly favored by Physician 1 and 4 (≈33.3%), while Physician 2 selected MP only once. The “Equivalent” rating was primarily assigned by Physicians 2 and 3 (≈33.3% and 40%, respectively).

A notable pattern emerges when analyzing the distribution of decisions between AP and MP across different plans: preference of MP was predominantly assigned to specific plans, such as plan 1, 6, 10, and 11. The planners responsible for these MP had a prior experience of more than 650 previously created plans. Conversely, for plans 2, 12, 13, and 14, the physicians consistently preferred the AP. In these cases, the average prior experience of the respective planners was only 275.5 plans. To further investigate this relationship, an analysis was conducted to assess the correlation between the planner's total experience measured by the number of previously created plans (see Table 2) and the physicians' evaluations. The results reveal a significant negative association (Spearman's ρ = –0.541, p = 0.037), suggesting that APs are preferred compared to MPs created by less experienced planners.

The overall inter‐physician agreement yielded a Fleiss’ kappa value of 0.259, indicating fair agreement among the four raters. This suggests a moderate level of variability in their assessments, highlighting the inherent challenges of subjective evaluation in treatment planning.

### 3.3 Treatment delivery parameters and dosimetric verification

The comparison of γ‐pass rates (3 mm/3%), MUs per fraction, MCS and LT between APs and MPs is summarized in Table 4. The γ‐passing rate was significantly higher for APs (98.40 ± 1.19%) compared to MPs (96.87 ± 3.37%, p = 0.02). For MUs, APs showed a slightly lower mean (627.06 ± 114.37 MU) compared to MPs (689.57 ± 188.93 MU); however, this difference was not statistically significant (p = 0.17). The MCS was comparable between APs (0.29 ± 0.04) and MPs (0.28 ± 0.07), with no significant difference (p = 0.44), indicating similar modulation characteristics between both plan types. Similarly, Leaf Travel (LT) was slightly lower for APs (393.79 ± 137.25) compared to MPs (419.09 ± 157.11), though this difference did not reach statistical significance (p = 0.11).

**TABLE 4:** Comparison of γ‐passing rates (3 mm/3%), total number of MUs per fraction, MCS and LT between automatically and manually created treatment plans.

	APs (Mean ± SD)	MPs (Mean ± SD)	p‐Values
γ‐passing rate (3 mm/3%)	98.40 ± 1.19	96.87 ± 3.37	0.02
MUs (MU)	627.06 ± 114.37	689.57 ± 188.93	0.17
MCS	0.29 ± 0.04	0.28 ± 0.07	0.44
LT	393.79 ± 137.25	419.09 ± 157.11	0.11

*Note*: Values are reported as mean ± standard deviation (SD). The *p*‐values indicate statistical significance between the two groups. The γ‐passing rate is significantly higher for automatically created plans (*p* = 0.02).

## 4 DISCUSSION

Manual treatment planning is a labor‐intensive process, especially in busy clinical routine, and poses significant challenges for inexperienced planners. The quality of manually created plans can vary considerably depending on the planner's level of experience, making it difficult to establish a consistently high standard. Developing a clinically acceptable treatment plan requires experienced planners to balance a multitude of competing parameters and tailor planning objectives to individual patient needs. To address these challenges, enhance efficiency, and ensure consistently high‐quality treatment planning, this study introduced UKER‐ATP, a novel automated planning tool. Designed specifically for VMAT lung tumor treatment planning, UKER‐ATP integrates the strengths of knowledge‐based and scripted‐based algorithms. It features functionalities such as dose prediction, auxiliary structure generation, adaptive objective adjustment, and plan quality evaluation, making it a robust and versatile tool for managing complex clinical scenarios even for experienced planners. The current version of UKER‐ATP was trained on a dataset using a conventional fractionation scheme of 50.4 Gy in 28 fractions. However, the framework is inherently adaptable and, with appropriate training data, can be retrained for alternative fractionation schemes and prescription doses, including boost plans, provided the training dataset reflects the corresponding prescriptions.

For the analysis, a cohort of 15 patients treated with VMAT was included in this study. These cases involved complex target‐OAR relationships, presenting some of the most challenging problems for plan optimization.

The dosimetric analysis suggests that APs generated by UKER‐ATP provide comparable or superior results to MPs. APs achieved lower $D_{2\%}$ and $D_{50\%}$ for the PTV, indicating reduced high‐dose exposure while maintaining coverage and improving dose uniformity. Conformity remained similar between both approaches. For OARs, APs significantly reduced mean doses and high‐dose volumes, particularly for the lungs, heart, and esophagus. The lower mean lung dose and lung $V_{20\;\mathrm{Gy}}$ are especially important for minimizing pulmonary toxicity,^37^ while improved heart and esophageal sparing highlight the potential of APs to reduce treatment‐related morbidity. Although CIs were generally comparable, APs tended to show slightly higher values than MPs, indicating a closer match between the target volume and the total volume covered by the reference isodose.

A key aspect of this study was the clinical evaluation of treatment plans by four physicians, which revealed notable inter‐observer variability. While automated plans were generally preferred, individual assessments varied significantly, highlighting the inherently subjective nature of plan evaluation. Beyond personal preferences, these differences also reflect distinct clinical workflows and underlying schools of thought. Physicians may prioritize different aspects of plan quality, such as target coverage, organ‐at‐risk sparing, or dose homogeneity, depending on their experience and clinical focus. A striking example of this divergence was the assessment of dose exposure to the contralateral lung: certain approaches placed greater emphasis on minimizing its dose, while others prioritized overall lung sparing. These variations underscore the challenge of aligning standardized algorithms with individualized decision‐making in clinical practice.

However, the relatively small evaluation cohort and fair inter‐physician agreement should be acknowledged as limitation. This limited agreement highlights the complexity of objectively assessing plan quality and emphasizes the need for broader, more standardized evaluation criteria in future studies.

Additionally, differing clinical focuses further contributed to the observed variability. Physician 3, whose primary focus was on sparing the lung, most frequently selected APs as the preferred option. Notably, UKER‐ATP was developed with Physician 3′s input, reflecting their specific clinical priorities in the system's design. On the other hand, Physicians 1 and 4 emphasized sparing the esophagus, including efforts to reduce both high‐dose and low‐dose exposures to mitigate long‐term toxicity. Importantly, sparing of the contralateral lung was also a key priority for Physicians 1 and 4, whereas Planners 2 and 3 placed greater emphasis on overall lung sparing.

The clinical analysis supports the observed correlation between planner experience and the preference for automated plans (AP). The results show that manual plans (MP) were predominantly chosen for specific cases (e.g., plan 1, 6, 10, and 11), which were created by planners with extensive experience. In contrast, APs were consistently favored for cases (e.g., plan 2, 12, 13, and 14) where the responsible MP planners had significantly less experience. This trend is further reinforced by the significant negative correlation, indicating that MPs from less experienced planners were more likely to be outperformed by APs. This finding aligns with the notion that manual planning does not always reach its full potential, particularly under time constraints or high workloads. The variability in planner expertise makes it difficult to ensure consistently high‐quality manual treatment planning. In this context, automated systems offer a stable baseline quality, particularly when planner experience varies. Moreover, they serve as useful reference tools for less experienced planners, providing insight into achievable treatment quality under given conditions. As a result, automation not only supports standardization but also facilitates learning and improvement in clinical practice.

To address inter‐observer variability and accommodate these differing priorities, UKER‐ATP incorporates a unique feature in the setup module that allows for dynamic customization of planning goals. This feature enables clinicians to adjust priorities between target coverage and OAR sparing in real‐time, ensuring that the system remains adaptable to various clinical preferences of the physicians. Immediate feedback on the impact of these adjustments enhances transparency and facilitates collaborative decision‐making. This structured approach standardizes the planning process while maintaining flexibility. By integrating this functionality directly into the planning process, UKER‐ATP not only ensures high‐quality, patient‐specific plans but also streamlines workflows, reducing the time required for iterative modifications.

The treatment delivery evaluation demonstrated that APs achieved significantly higher γ‐passing rates (3 mm/3%) compared to MPs, highlighting superior plan deliverability and robustness. While the difference in MUs was not statistically significant, there was a tendency for APs to exhibit slightly lower MUs, suggesting potential efficiency gains in treatment delivery. Increased plan complexity has been linked to challenges in radiation delivery,^38^ as higher MUs are often associated with more complex plans. However, contrary to findings from other studies,^25^, ^39^, ^40^, ^41^ no significant increase in MUs was observed in the AP cases of this study, despite those earlier studies focusing on different tumor sites, not lung tumors. With UKER‐ATP, APs tended to require fewer MUs while achieving comparable PTV coverage and superior sparing of OARs compared to MPs. A similar trend was observed by Lou et al.^25^ in cases of esophageal carcinoma, where reduced MUs were attributed to improved beam angle optimization—an aspect not considered in this study. Here, the tendency toward lower MUs might be explained by constraints implemented in RayStation during the plan setup and planning goal adjustment phases. In addition to MUs, LT and MCS were analyzed to further assess plan complexity and deliverability. While LT was slightly lower for APs compared to MPs, the difference was not statistically significant. Reduced LT may indicate more efficient and potentially smoother MLC movement. Similarly, MCS values were comparable between APs and MPs, indicating that both plan types exhibited similar modulation characteristics.

Several prior studies have highlighted that exceptional efficiency is a defining trait of automated planning,^25^, ^40^, ^42^, ^43^ a conclusion that aligns with our results. While this specific aspect has not been thoroughly investigated, the time actively spent interacting with the treatment planning system has been reduced to 15 min or less. Additional analyses are planned to explore this aspect in greater detail.

Despite the promising results, this study has some limitations that warrant further investigation. The dose prediction model used in this work is based on a knowledge‐based method. While the knowledge‐based approach applied in this study has proven robust and interpretable, it does not enable the direct prediction of 3D dose distributions. Such predictions, as facilitated by recent deep neural network‐based methods, may further improve the generation of fully automated treatment plans. Additionally, this study did not include collimator angle and beam angle optimization algorithms, which can improve plan quality by optimizing beam modulation and sparing critical structures. Furthermore, non‐coplanar approaches^44^ should be examined, as well‐chosen beam angles can lead to further reductions in lung and heart doses while maintaining consistent target coverage.

UKER‐ATP itself is currently implemented as an in‐house scripting solution within RayStation Version A12 and was developed solely for research purposes. However, the code can be shared upon request.

## 5 CONCLUSION

In this study, we introduced a novel automated planning approach called UKER‐ATP, which integrates the advantages of knowledge‐based and script‐based planning algorithms. We demonstrated that UKER‐ATP generates treatment plans with dose distributions comparable to or better than manual planning while reducing plan complexity and saving time. These findings highlight the potential of UKER‐ATP to streamline the radiotherapy treatment planning process and elevate the overall quality of treatment plans.

## AUTHOR CONTRIBUTION

**Johann Brand**: Conceptualization; methodology; software; formal analysis; writing—original draft; visualization. **Juliane Szkitsak**: Conceptualization; supervision; writing—review and editing; visualization. **Bernd‐Niklas Axer**: Methodology; formal analysis. **Lucas Pieper**: Methodology; formal analysis. **Oliver J. Ott**: Resources; investigation; validation. **Marlen Haderlein**: Investigation; validation; writing—review; and editing. **Florian Putz**: Investigation; validation. **Rainer Fietkau**: Investigation; validation; writing—review; and editing. **Christoph Bert**: Supervision; writing—review; and editing. **Stefan Speer**: Conceptualization; supervision; writing—review; and editing.

## CONFLICT OF INTEREST STATEMENT

The authors declare no conflicts of interest.

## Data Availability

The data that support the findings of this study are available from the corresponding author upon reasonable request.

## References

[1] Bortfeld TR , Kahler DL , Waldron TJ , Boyer AL . X‐ray field compensation with multileaf collimators. Int J Radiat Oncol Biol Phys. 1994;28(3):723‐730. doi:10.1016/0360-3016(94)90200-3 8113118

[2] Brahme A . Optimization of stationary and moving beam radiation therapy techniques. Radiother Oncol. 1988;12(2):129‐140. doi:10.1016/0167-8140(88)90167-3 3406458

[3] Convery DJ , Rosenbloom ME . The generation of intensity‐modulated fields for conformal radiotherapy by dynamic collimation. Phys Med Biol. 1992;37(6):1359. doi:10.1088/0031-9155/37/6/012

[4] Yu CX . Intensity‐modulated arc therapy with dynamic multileaf collimation: an alternative to tomotherapy. Phys Med Biol. 1995;40(9):1435. doi:10.1088/0031-9155/40/9/004 8532757

[5] Otto K . Volumetric modulated arc therapy: iMRT in a single gantry arc. Med Phys. 2008;35(1):310‐317. doi:10.1118/1.2818738 18293586

[6] Cao D , Afghan MKN , Ye J , Chen F , Shepard DM . A generalized inverse planning tool for volumetric‐modulated arc therapy. Phys Med Biol. 2009;54(21):6725. doi:10.1088/0031-9155/54/21/018 19841516

[7] Hunte SO , Clark CH , Zyuzikov N , Nisbet A . Volumetric modulated arc therapy (VMAT): a review of clinical outcomes‐what is the clinical evidence for the most effective implementation?. Br J Radiol. 2022;95(1136):20201289. doi:10.1259/bjr.20201289 35616646 PMC10162061

[8] Nguyen D , Lin MH , Sher D , Lu W , Jia X , Jiang S . Advances in automated treatment planning. Semin Radiat Oncol. 2022;32(4):343‐350. doi:10.1016/j.semradonc.2022.06.004 36202437 PMC9851906

[9] Speer S , Klein A , Kober L , Weiss A , Yohannes I , Bert C . Automation of radiation treatment planning : evaluation of head and neck cancer patient plans created by the Pinnacle(3) scripting and auto‐planning functions. Strahlenther Onkol. 2017;193(8):656‐665. doi:10.1007/s00066-017-1150-9 28653120

[10] Wang J , Hu J , Song Y , et al. VMAT dose prediction in radiotherapy by using progressive refinement UNet. Neurocomputing. 2022;488:528‐539. doi:10.1016/j.neucom.2021.11.061

[11] Chung HT , Lee B , Park E , Lu JJ , Xia P . Can All centers plan intensity‐modulated radiotherapy (IMRT) effectively? An external audit of dosimetric comparisons between three‐dimensional conformal radiotherapy and IMRT for adjuvant chemoradiation for gastric cancer. Int J Radiat Oncol Biol Phys. 2008;71(4):1167‐1174. doi:10.1016/j.ijrobp.2007.11.040 18234440

[12] Hirotaki K , Tomizawa K , Moriya S , et al. Fully automated volumetric modulated arc therapy planning for locally advanced rectal cancer: feasibility and efficiency. Radiat Oncol. 2023;18(1):147. doi:10.1186/s13014-023-02334-0 37670390 PMC10481560

[13] Wu H , Jiang F , Yue H , Zhang H , Wang K , Zhang Y . Applying a RapidPlan model trained on a technique and orientation to another: a feasibility and dosimetric evaluation. Radiat Oncol. 2016;11(1):108. doi:10.1186/s13014-016-0684-9 27538431 PMC4990878

[14] Wu H , Jiang F , Yue H , Li S , Zhang Y . A dosimetric evaluation of knowledge‐based VMAT planning with simultaneous integrated boosting for rectal cancer patients. J Appl Clin Med Phys. 2016;17(6):78‐85. doi:10.1120/jacmp.v17i6.6410 27929483 PMC5690500

[15] Zhang H , Yu Y , Zhang F . Prediction of dose distributions for non‐small cell lung cancer patients using MHA‐ResUNet. Med Phys. 2024;51(10):7345‐7355. doi:10.1002/mp.17308 39024495

[16] Kandalan RN , Nguyen D , Rezaeian NH , et al. Dose prediction with deep learning for prostate cancer radiation therapy: model adaptation to different treatment planning practices. Radiother Oncol. 2020;153:228‐235. doi:10.1016/j.radonc.2020.10.027 33098927 PMC7908143

[17] Putz F , Fietkau R . The increasing role of artificial intelligence in radiation oncology: how should we navigate it?. Strahlenther Onkol. 2025;201(3):207‐209. doi:10.1007/s00066-025-02381-4 39971777 PMC11839879

[18] Shao Y , Guo J , Wang J , et al. Novel in‐house knowledge‐based automated planning system for lung cancer treated with intensity‐modulated radiotherapy. Strahlenther Onkol. 2024;200(11):967‐982. doi:10.1007/s00066-023-02126-1 37603050 PMC11527916

[19] Song Y , Wang Q , Jiang X , Liu S , Zhang Y , Bai S . Fully automatic volumetric modulated arc therapy plan generation for rectal cancer. Radiother Oncol. 2016;119(3):531‐536. doi:10.1016/j.radonc.2016.04.010 27131593

[20] Hazell I , Bzdusek K , Kumar P , et al. Automatic planning of head and neck treatment plans. J Appl Clin Med Phys. 2016;17(1):272‐282. doi:10.1120/jacmp.v17i1.5901 26894364 PMC5690191

[21] Lu L , Sheng Y , Donaghue J , et al. Three IMRT advanced planning tools: a multi‐institutional side‐by‐side comparison. J Appl Clin Med Phys. 2019;20(8):65‐77. doi:10.1002/acm2.12679 31364798 PMC6698808

[22] Rønde HS , Wee L , Pløen J , Appelt AL . Feasibility of preference‐driven radiotherapy dose treatment planning to support shared decision making in anal cancer. Acta Oncol (Madr). 2017;56(10):1277‐1285. doi:10.1080/0284186X.2017.1315174 28447539

[23] Ghandour S , Matzinger O , Pachoud M . Volumetric‐modulated arc therapy planning using multicriteria optimization for localized prostate cancer. J Appl Clin Med Phys. 2015;16(3):5410. doi:10.1120/jacmp.v16i3.5410 26103500 PMC5690115

[24] Gleeson I , Bolger N , Chun H , et al. Implementation of automated personalised breast radiotherapy planning techniques with scripting in Raystation. Br J Radiol. 2023;96(1144):20220707. doi:10.1259/bjr.20220707 36728760 PMC10078863

[25] Lou Z , Cheng C , Mao R , et al. A novel automated planning approach for multi‐anatomical sites cancer in Raystation treatment planning system. Physica Med. 2023;109:102586. doi:10.1016/j.ejmp.2023.102586 37062102

[26] Hong L , Huang Q , Zhou Y , et al. Automated and clinical‐criteria‐driven planning for lung cancer using the expedited constrained hierarchical optimization (ECHO) system. Int J Radiat Oncol Biol Phys. 2022;114(3):e583. doi:10.1016/j.ijrobp.2022.07.2256

[27] Fjellanger K , Hordnes M , Sandvik IM , et al. Improving knowledge‐based treatment planning for lung cancer radiotherapy with automatic multi‐criteria optimized training plans. Acta Oncol. 2023;62(10):1194‐1200. doi:10.1080/0284186X.2023.2238882 37589124

[28] Brand J , Szkitsak J , Ott JO , Bert C , Stefan S . An extension to the OVH concept for knowledge‐based dose volume histogram prediction in lung tumor volumetric‐modulated arc therapy. J Appl Clini Med Phys. 2025;26(6):e70090. doi:10.1002/acm2.70090 PMC12148784 40181212

[29] Wu B , Ricchetti F , Sanguineti G , et al. Patient geometry‐driven information retrieval for IMRT treatment plan quality control. Med Phys. 2009;36(12):5497‐5505. doi:10.1118/1.3253464 20095262

[30] Kataria T , Sharma K , Subramani V , Karrthick KP , Bisht SS . Homogeneity index: an objective tool for assessment of conformal radiation treatments. J Med Phys. 2012;37(4):207–213. doi:10.4103/0971-6203.103606 23293452 PMC3532749

[31] Feuvret L , Noël G , Mazeron J‐J , Bey P . Conformity index: a review. Int J Radiat Oncol Biol Phys. 2006;64(2):333‐342. doi:10.1016/j.ijrobp.2005.09.028 16414369

[32] Li G , Zhang Y , Jiang X , et al. Evaluation of the ArcCHECK QA system for IMRT and VMAT verification. Physica Med. 2013;29(3):295‐303. doi:10.1016/j.ejmp.2012.04.005 22583979

[33] Younge KC , Matuszak MM , Moran JM , McShan DL , Fraass BA , Roberts DA . Penalization of aperture complexity in inversely planned volumetric modulated arc therapy. Med Phys. 2012;39(11):7160‐7170. doi:10.1118/1.4762566 23127107 PMC3505204

[34] Masi L , Doro R , Favuzza V , Cipressi S , Livi L . Impact of plan parameters on the dosimetric accuracy of volumetric modulated arc therapy. Med Phys. 2013;40(7):071718. doi:10.1118/1.4810969 23822422

[35] Cavinato S , Scaggion A , Paiusco M . Technical note: A software tool to extract complexity metrics from radiotherapy treatment plans. Med Phys. 2024;51(11):8602‐8612. doi:10.1002/mp.17365 39186793

[36] Cavinato S , Scaggion A . UCoMX: Universal Complexity Metrics Extractor . Version (1.01). Zenodo; 2024. doi:10.5281/zenodo.8276837

[37] Marks LB , Bentzen SM , Deasy JO , et al. Radiation dose‐volume effects in the lung. Int J Radiat Oncol Biol Phys. 2010;76(3):S70‐6. doi:10.1016/j.ijrobp.2009.06.091 Suppl 20171521 10.1016/j.ijrobp.2009.06.091 PMC3576042

[38] Wall PDH , Fontenot JD . Evaluation of complexity and deliverability of prostate cancer treatment plans designed with a knowledge‐based VMAT planning technique. J Appl Clin Med Phys. 2020;21(1):69‐77. doi:10.1002/acm2.12790 10.1002/acm2.12790 PMC6964749 31816175

[39] Cilla S , Ianiro A , Romano C , et al. Template‐based automation of treatment planning in advanced radiotherapy: a comprehensive dosimetric and clinical evaluation. Sci Rep. 2020;10(1):423. doi:10.1038/s41598‐019‐56966‐y 31949178 10.1038/s41598-019-56966-y PMC6965209

[40] Cilla S , Romano C , Morabito VE , et al. Personalized treatment planning automation in prostate cancer radiation oncology: a comprehensive dosimetric study. original research. Front Oncol. 2021;11:636529. doi:10.3389/fonc.2021.636529 34141608 PMC8204695

[41] Buschmann M , Sharfo AWM , Penninkhof J , et al. Automated volumetric modulated arc therapy planning for whole pelvic prostate radiotherapy. Strahlenther Onkol. 2018;194(4):333‐342. doi:10.1007/s00066‐017‐1246‐2 29270648 10.1007/s00066-017-1246-2 PMC5869893

[42] Yang Y , Shao K , Zhang J , Chen M , Chen Y , Shan G . Automatic planning for nasopharyngeal carcinoma based on progressive optimization in RayStation treatment planning system. Technol Cancer Res Treat. 2020;19:1533033820915710. doi:10.1177/1533033820915710 32552600 10.1177/1533033820915710 PMC7307279

[43] Sheng Y , Li T , Yoo S , et al. Automatic planning of whole breast radiation therapy using machine learning models. original research. Front Oncol. 2019;9:750. doi:10.3389/fonc.2019.00750 31440474 PMC6693433

[44] Fleckenstein J , Boda‐Heggemann J , Siebenlist K , et al. Non‐coplanar VMAT combined with non‐uniform dose prescription markedly reduces lung dose in breath‐hold lung SBRT. Strahlenther Onkol. 2018;194(9):815‐823. doi:10.1007/s00066‐018‐1316‐0 29802434 10.1007/s00066-018-1316-0

